# Supplementary material for: Diagnostic and prognostic significance of premature ventricular complexes in community and hospital-based participants: A scoping review
Source: PLoS One. 2021 Dec 23;16(12):e0261712. doi: 10.1371/journal.pone.0261712 (PMC8699640; doi:10.1371/journal.pone.0261712)
Supplement: S2 Table — (DOCX) [file pone.0261712.s002.docx]

**S2 Table. Database search results.**

| **Database** | **Query** | **Results** |
| --- | --- | --- |
| CINAHL | (MH "Premature Ventricular Contractions")  *Filters: Abstract Available, Academic Journals, English.* | 803 |
| Embase | ('heart ventricle extrasystole'/exp OR 'heart ventricle extrasystole') AND ([article]/lim OR [article in press]/lim) AND [english]/lim AND ([young adult]/lim OR [adult]/lim OR [middle aged]/lim OR [aged]/lim OR [very elderly]/lim) AND [clinical study]/lim | 3,696 |
| PubMed | premature ventricular contraction[MeSH]  *Filters: Humans, English.* | 1,750 |
| Web of Science Core Collection | ((premature ventricular contraction) OR (ventricular extrasystole) OR (ventricular extra systole) OR (premature ventricular beat))  *Filters: Document types (Articles), English.* | 3,814 |
