# Supplementary material for: Diagnostic and prognostic significance of premature ventricular complexes in community and hospital-based participants: A scoping review
Source: PLoS One. 2021 Dec 23;16(12):e0261712. doi: 10.1371/journal.pone.0261712 (PMC8699640; doi:10.1371/journal.pone.0261712)
Supplement: S3 Table — (DOCX) [file pone.0261712.s003.docx]

**S3 Table. Methodology and key findings of the included studies.**

| **Authors** | **Study Aim** | **Sample Criteria** | **Follow-Up Period** | **Analysis Method** | **Variables in Multivariate/Max Adjusted Models** | **Key Findings** |
| --- | --- | --- | --- | --- | --- | --- |
| Chiang 1969 ^33^ | Determine the relationship of premature beats to the subsequent morbidity and mortality from CHD.  Outcome variable: | General population with premature systoles on the ECG recording (Tecumseh Community Health Study) | 6 year | Chi-square | Not provided | Incidence rate of sudden death among those with preexistent PVCs was higher compared to those without PVCs |
| Desai 1973^34^ | Define the effect on mortality of PVC present in a routine ECG and if there were different risks in the various subgroups within the total PVC population. | One or more PVC on a standard 12-lead ECGs | 3.5 years (3 - 4 years) | Not provided | Not provided | - The ratio of deaths in the PVC group compared to the control group was 1.6 to 1 - In patient with CVD, mortality rate was higher among those with PVCs compared to those without PVCs |
| Ruberman 1977 ^35^ | Assess the role of PVCs in influencing mortality of coronary patients. | Male, insured, had at least one MI or angina | Up to 4 years (mean 24.4 months) | Peto-Pike chi-square; Cox’s regression | Age ≥65, heart rate ≥90, duration of heart disease, treatment with diuretics, ST-segment depression, CHF, and complex PVCs | - Presence of baseline complex PVCs was associated with higher risk of SCD (RR 2.8) and all-cause death (RR 1.9) - At 3-years, the cumulative probability for SCD was higher among men with R-on-T or runs of PVCs compared to men with bigeminy or multiform PVCs (RR 2.3) |
| Schulze 1977 ^83^ | Evaluate the relative role of PVCs and depressed LVEF following AMI in predicting mortality. | Discharged from coronary care unit and were diagnosed with AMI. Exclusion: MI occurred as a complication of surgery or catheterization; documented malignancy or end-stage renal or hepatic disease; technical problems made completion of the study not feasible | 2 - 16 months (mean 7 months) | Unpaired t-test/chi-square; Life Table survival analysis | Not provided | Life table survival analysis showed that patients with PVC class III – V (Lown criteria) had a higher sudden death rate compared to those with PVC class 0 – II |
| Boudoulas 1979 ^36^ | Define the prevalence of PVCs in ambulatory patients and the characteristics of PVCs predisposing to VT or VF. | More than “occasional” PVCs (>1 /min) detected by exercise testing or 24-hr monitoring, who were suspected to have dysrhythmias and were referred for ambulatory monitoring and exercise testing to define the nature of dysrhythmias | NR | Student t-test; chi-square | Not provided | - The frequency of VT/VF, or both, was greater in patients with late-PVCs compared with early- and midcycle-PVCs. - Patients with frequent multiform PVCs had a greater frequency of VT or VF compared with patients with uniformed PVCs. |
| Moss  1979 ^84^ | Explore the role of both simple and complex PVCs as they relate to sudden and non-sudden cardiac death throughout both the early and late posthospital phases of AMI. | Younger than 66 years of age who entered coronary care units with either definite or probable AMI and who survived hospitalization | Average 36 months (1-60 months) | Wilcoxon-Breslow; Mantel-Haenszel | Age >55; education <10th grade; social class IV and V; history of cigarette smoking; NYHA class II, III, IV; history of angina; history of MI; posterior MI; anterior MI; LV dysfunction | Complex PVCs were associated with increased cardiac death rate; but did not discriminate sudden from non-sudden death |
| Rengo 1979 ^85^ | Assess if ECG patterns of PVCs were related to an increased risk of sudden death. | Patients with one or more PVCs on standard 12-lead ECG at hospital admission | 30 months | Chi-square | Not provided | Patients with PVC coupling interval of <360 msec. had a higher rate of sudden death compared to patients with coupling interval of >360 msec. |
| Sclarovsky 1979 ^86^ | Investigate the relationship between the ECG location of the AMI and the origin of the PVCs, and to study their prognosis in relation to serious arrhythmias. | Definative AMI in whom the diagnosis of the origin of PVCs was obtained. Exclusion: receiving digitalis, beta-blockers, anti-arrhythmic drugs, diuretics, and those with electrolyte disturbances | NR | Chi-square; Fisher’s exact | Not provided | The origin of acute PVCs was highly correlated with the location of AMI |
| Lichtenberg 1980 ^87^ | Determine the validity of the PVC QR pattern in the diagnosis of MI. | Patients undergoing cardiac catheterization | NR | Chi-square | Not provided | - PVC in V_1_ with a Q ≥0.04 sec and a Q/R ratio >0.1 had a greater applicability in the detection of anterior MI (sens 0.36, spec 0.87, and predictive value 0.67). - Presence of a wide Q PVC of ≥0.02 sec in aVF was correlated with abnormal inferior wall motion (sens 0.22, spec 0.94, predictive value 0.70) |
| Rabkin 1981 ^37^ | Examine the relationship between the occurrence and characteristics of PVCs and subsequent development of IHD in men without clinically apparent heart disease. | Individuals from community setting without clinical manifestation of IHD who had ventricular ectopic beats | 10.8 ± 0.4 (± 1 SEM) | Chi-square; Fisher’s exact; Life Table method | Age groups | Presence of PVCs was correlated with patient outcomes: total IHD (RR 1.80), MI (RR 1.43), sudden death (RR 4.18), coronary insufficiency or suspected MI (RR 1.93), and angina pectoris (RR 1.41) |
| Dash  1983 ^88^ | Evaluate the value of VPB analysis in the diagnosis of prior MI. | Patients with diagnostic cardiac catheterization and had PVCs on ECGs recorded the day before procedure | NR | Chi-square with Yate’s correction | Not provided | - Morphologic analysis of PVCs had a low sensitivity (29%) but high specificity (97%) and predictive value (86%) for the diagnosis of MI - Sinus beats analysis had higher sensitivity (52%) than PVC morphology, but with similar specificity (97%) and predictive value (92%) |
| Bigger 1984 ^89^ | Define the interrelationships of frequency and repetitiveness of PVCs, LV dysfunction, and mortality. | Positive for acute myocardial ischemia <70 years of age. Exclusion: life-threatening comorbities; lived too far away from participating centers for follow up | Average 22 months (12 - 36 months) | Laird-Oliver survivorship analysis | LVEF, PVC frequency categories, and PVC repetitiveness categories | - Patients with PVC ≥3/hr had a higher risk for dying compared to those with PVC <1/hr (HR 2) - Patients with run PVC had a higher risk for dying compared to those with no repetitive PVC (HR 1.9) - ≥3 PVCs/hr was an independent predictor of higher risk for dying >6 months of MI (HR 3.7 vs HR 1.2 ≤6 months) |
| Abdalla 1987 ^38^ | Examine the relation between PVCs and subsequent SCD in apparently healthy middle-aged men (part of the Multiple Risk Factor Intervention Trial [MRFIT]). | Apparently healthy patients included in the MRFIT without previous history of MI or DM, or ECG abnormalities other than PVCs | 7.5 years | Cox proportional hazard | Age, SBP, serum cholesterol level, and cigarette smoking | Presence of any PVC (RR 3.0 [95% CI 1.3-7.3]), frequent PVCs (RR 3.8 [95% CI 1.4-10.9]), and frequent/complex PVCs (RR 4.2 [95% CI 1.6-10.7]) were significantly associated with SCD |
| Kostis  1987 ^90^ | Define the usefulness of various definitions of ventricular ectopic activity in predicting the mortality rate among survivors of AMI. | Definitive AMI who had 24 hour ambulatory ECG 2 to 21 days after admission to a coronary care unit. Exclusion: history of severe CHF, bronchial asthma as an adult, marked bradycardia and life-threatening illness other than CAD, those who had or were likely to have aortocoronary bypass surgery and those who were taking or were likely to take a beta-adrenergic blocking agent (part of Beta Blocker Heart Attack Trial, BHAT) | 25 months (12 to 40 months) | Student’s t-test and logistic regression | Age, employed, current cigarette smoker, prior MI, diastolic BP, systolic BP, heart rate, hematocrit, serum cholesterol, elevated LDH, anterior infarction, ST depression, major LV hypertrophy, cardiothoracic ratio, experienced complications during hospitalization, history of CHF, history of diabetes, beta-blocker use prior to the study period, antiplatelet use at entry, digitalis use at entry, antiarrhythmic drug use at entry, Holter characteristics from 24-hour Holter | - Ventricular ectopic activities were significant predictors of mortality (adjusted OR ranging from 1.003 to 2.23) - Presence of multiform PVC was an independent predictor for atherosclerotic sudden death (OR 2.43) - Presence of a mean PVC ≥10/h was independently associated with atherosclerotic non-sudden death (OR 2.25)   *NOTE:* Ventricular ectopic activities include PVC/hr, couplet/hr, and presence of mean PVC/hr >0, mean PVC/hr ≥10, and multiform PVCs. |
| Minisi 1988 ^91^ | Examine association between the extent of CAD and frequency of PVCs in survivors of MI. | 6 days after MI. Exclusion: >74 years, women with childbearing potential, malignancies, significant and/or potentially lethal organ system disorders (other than cardiac), severe hypertension (SBP ≥180 mm Hg or DBP ≥120 mm Hg), cardiogenic shock, significant non-CAD, glaucoma, and symptomatic urinary tract obstruction in males | NR | Ordinal regression analysis | Not provided | - Both PVC frequency and LVEF were independently important predictors of CAD severity by vessels stenosis and jeopardy score - The median PVC frequency was 1/hr, 0.6/hr, and 6/hr in zero to one-, two-, and three-vessel CAD, respectively (zero to one- and two-V CAD vs three-V CAD *p* <0.01, one-V CAD vs two-V CAD *p*=NS) |
| Rasmussen 1988 ^92^ | Define patients at increased risk of death in the early phase after MI based on ventricular ectopic activity from 24-hour ECG recordings. | Patients surviving initial stage of AMI. Exclusion: atrial fibrillation and chronic treatment for ventricular dysrhythmias | 20 - 27 months | Wald test; Kaplan-Meier survival analysis | Age, presence of PVC, and HF | - All-cause mortality was significantly higher in the group with than in that without complex PVCs - Complex PVCs were associated with increased risk of mortality (relative risks 3.6) |
| Moulton 1990 ^93^ | Characterize the relationship between PVC morphology and myocardial disease state. | Consecutive patients undergoing cardiac catheterization, echocardiography, or nuclear ventriculography; had at least 1 PVC present on 12-lead ECG | NR |  | Not provided | - Patients with type II PVCs had significantly higher EDVI and lower EF compared to patients with type I PVC - PVC QRS morphology (type I vs type II) could classify EDVI (> or ≤90 ml/m2) and LVEF (< or ≥50%), with sen 92%, spec 80%, and accuracy of 86%   *NOTE:* Type I PVC: QRS complexes with either smooth and uninterrupted contour or narrow (<40 msec) notches.  Type II PVC: QRS that is wide (≥40 msec) notches or shelves. |
| Wilson 1992 ^94^ | Study the independent contribution of ventricular ectopic activity in predicting mortality (part of Beta Blocker Heart Attack Trial, BHAT) | Patient with definite AMI who had <2PVC/h. Exclusion: history of severe CHF, marked bradycardia, life-threatening illnesses other than CAD, those who had bronchial asthma as adults, those who had or were likely to have aortocoronary bypass surgery, patients taking or were likely to take beta blockers | 25 months (range 11 - 40 months) | Logistic regression | Age, employment status, smoking, prior MI, diastolic BP, hematocrit, elevated LDH, ST depression (on ECG), cardiothoracic ratio (from x-ray film), hospital complications, CRF, diabetes, beta-blocker use, anti-platelet use, antiarrhythmic drug use | Patients with an average of 0.5 or more but <1PVC/h had significant increase in mortality (190%) than patients who had no PVCs, and by 95% compare to patients with <0.5 PVC/h (p<0.001) |
| Fujimoto 1994 ^95^ | Clarify the relationship between the characteristics of PVCs and the patients’ prognosis. | Patients with frequent PVCs (>1,000 beats/day). Exclusion: patients with acute heart disease (acute MI, myocarditis or pericarditis) or sustained ventricular tachycardia | 4 years | One-way ANOVA; unpaired t-test; chi-square | Not provided | - Lown grades 4a and 4b were more frequent in patients with underlying diseases who survived and those with cardiac death, compared to patients with idiopathic PVC and survived (p<0.05) - The mean coupling interval was significantly longer in those who suffered cardiac death as compared to patients with idiopathic PVC who survived |
| Statters 1996 ^96^ | Compare the predictive value of PVCs in survivors of AMI who received or did not receive thrombolytic therapy. | Admitted to coronary care unit and diagnosed with AMI. Exclusion: noncardiac diseases likely to influence mortality, important nonischemic cardiac disease, or a history of cardiac surgery or permanent pacemaker | 1 year | Kaplan-Meier; Wilcoxon-Breslow | Not provided | The highest PVC frequency by high- and low-risk groups for mortality was 25 VPCs/hour after thrombolysis, but 10 VPCs/hour for patients without thrombolysis |
| Dabrowski 1998 ^39^ | Determine whether the evaluation of PVC-QT dispersion (QTd-V) could identify patients at high risk of subsequent arrhythmic events. | Had at least one PVC on routine 12-lead ECG prior to 24-h Holter recording, complained of syncope, dizziness, palpitations, and angina | 26 ± 19 months | Kaplan-Meier; cox proportional hazard | QT dispersion in PVCs ≥100 ms, LVEF <40%, underlying heart disease, QRS duration of PVCs >150msec, and complete bundle branch block | - PVC-QT dispersion (QTd-V) was greater in patients with arrhythmic events - QTd-V ≥ 100ms and LVEF < 40% were independent predictors of arrhythmic events   *NOTE:* Arrhythmic events: sustained ventricular tachycardia, ventricular fibrillation or sudden cardiac death. |
| Vaage-Nilsen 1998 ^97^ | Evaluate the prognostic implications of ventricular ectopy recorded by 24-h Holter monitoring (in conjunction with a randomized trial [Danish Verapamil Infarction Trial II, DAVIT II). | AMI <76 years of age. Exclusion: taking calcium antagonists or beta blockers for angina pectoris, hypertension, or arrhythmias; uncontrolled HF not controlled, taking furosemide, second- or third-degree AV block; hypotension or other severe diseases; permanent atrial fibrillation and treatment with antiarrhythmic drugs or digoxin | 615 – 1702 days | Kaplan-Meier; multivariate cox analysis | Age, previous AMI, history of angina pectoris, intermittent claudication, index AMI involving anterior and inferior or posterior wall, resting heart rate, left bundle-branch or IV block on ECG, and HF | >10 VPCs/hr for 1 week (HR 2.81, 95%CI 1.55-5.08) or 1 month (HR 3.13, CI 1.46-6.69) after acute MI independently predicted long-term mortality |
| Dabrowski 1999 ^40^ | Evaluate whether dispersion of repolarization calculated from VPBs on a 12-lead ECG offers improved risk stratification in patients after MI. | PVCs on a 12-lead ECG recorded at least 4 weeks post-MI | 38 ± 17 months | Cox proportional hazard | Age ≥60 years, complete bundle branch block, LVEF <40%, JT dispersion ≥100 msec, QT dispersion ≥100 msec, and R on T index of PVCs <1 | - QTd-V (HR 3.89), JTd-V (HR 2.77), and “R-on-T” index of PVCs<1 (HR 1.89) were predictors of mortality (univariate analysis) - QTd-V ≥100 msec was an independent predictor of mortality (HR= 3.1041; 95% CI= 1.7–9.4) |
| Dabrowski 2000 ^98^ | Assess prognostic significance of QTd-V and QTd-S in post MI patients with PVCs on a routine ECG. | Exhibited PVCs on a standard 12-lead ECG after MI , referred for 24-h ECG monitoring due to symptoms (syncope, dizziness, palpitations, and angina). Exclusion: treated with class I or III antiarrhythmic drugs at presentation, atrial fibrillation or flutter, complete BBB, or pacemaker rhythm, and patients with unmeasurable QT intervals from sinus beats or PVCs in > ECG lead | 35 ± 17 months | Kaplan-Meier; cox proportional hazard | Age ≥60 years, time between MI - follow up <5 mo, LVEF <40%, QTd-S ≥100 ms, QTd-V ≥100 ms. | - QTd-V and QTd-S were significantly greater in patients with arrhythmic events - Patients with QTd-V ≥100 msec had higher arrhythmic event rates compared with those with QTd-V <100 msec. - QTd-V ≥100 msec was an independent predictor of arrhythmic events (HR 4.27, 95%CI 2.4 - 11.1)   *NOTE:* Arrhythmic events: sustained VT, VF, or sudden cardiac death. |
| Jouven 2000 ^41^ | Assess long-term outcomes for asymptomatic persons with exercise-induced PVC. | Men aged 42 to 53 years with ECG, physical examination, exercise test, and free of CHD. Exclusion: Exercise testing exclusion: if individuals had known or suspected cardiovascular disease of any grade or cause, systolic BP >180 mmHg at rest, or any abnormality on standard 12-lead ECG at rest according to the Minnesota code | 23 years | ANOVA; logistic regression; cox proportional hazard | Age, body-mass index, heart rate at rest, systolic BP, tobacco use, level of physical activity, diabetes, total cholesterol level, and PVCs before exercise and during recovery from exercise | Occurrence of frequent PVC during exercise testing independently associated with an increased risk of death from cardiovascular causes (RR 2.53, 95% CI 1.65-3.88) |
| Hatanaka 2002 ^42^ | Evaluate the relationship between PVC patterns on routine ECG and heart diseases. | Individuals who underwent clinical examination for heart disease and had one or more PVCs on routine 12-lead ECG. Exclusion: atrial fibrillation (valvular heart disease, lone atrial fibrillation, ischemic heart disease, cardiomyopathy), conduction disturbance (AV block, LBBB, RBBB, Wolff-Parkinson-White syndrome), taking antiarrhythmic drugs or poorly recorded ECG tracing | NR | Chi-square with Bonferroni correction | Not provided | - Individuals with LBBB type PVC morphology without axis deviation had a lower incidence of having heart disease than those with LBBB morphology type PVC with LAD, RBBB morphology type PVC with LAD and without axis deviation - Asymptomatic individuals with the LBBB type PVC pattern without axis deviation had a lower incidence of heart disease than those with other PVC patterns |
| Fries  2003 ^101^ | Determine the clinical significance of the R-on-T phenomenon in patients with an ICD | Patients with ICD and had recorded spontaneous tachyarrhythmias | NR | Mann-Whitney U; chi-square | Not provided | R-on-T PVCs rarely induced sustained VT in patients with ICD. However, R-on-T VTs occurred particularly in patients with CAD and more often, the VTs are polymorphic. |
| Morshedi-Meibodi 2004 ^43^ | Assess prognostic impact of exercise-induced PVCs (EiPVCs) on a large, community-based sample of young to middle-aged men and women free of overt CVD. | Framingham Offspring Study participants who underwent routine treadmill test during their second examination (4 years after the initial examination). Exclusion: Individuals with any prevalent CVD, valvular heart disease, COPD, usage of cardiac glycoside or blocking agents, or presence of PVCs at rest | 15 years (SD not provided) | Cox proportional hazard | Age, hypertension, smoking, total/HDL cholesterol, diabetes, resting heart rate, peak heart rate, duration of exercise, and ischemic ST-segment response | - EiPVC was associated with increased all-cause mortality rates (adjusted hazard ratio, 1.86 95% CI 1.24-2.79 for infrequent EiPVCs, and 1.71, 95% CI 1.18-2.49 for frequent versus none) - Frequent EiPVC was associated with >3-fold increased risk for cardiovascular death (HR, 3.45; 95% CI 1.69 - 7.07) |
| Carrim 2005 ^44^ | Examine relationship between mean frequency of PVCs and a predisposition to malignant ventricular arrhythmias in a group of patients with an ICD device. | Implantable cardioverter defibrillation (ICD) device for underlying ischemic or non-ischemic cardiac pathology, with available single and run PVC counts | NR | Mann-Whitney U; chi-square | Not provided | In patients with IHD, the frequency of single-PVCs and run-PVCs were significantly higher in those with VT/VF than that without VT/VF |
| Massing 2006 ^45^ | Estimate the magnitude of PVC-associated risk for CHD and mortality among patients without a history or clinical evidence of CHD at the time PVCs were detected. | Participants from general population (ARIC Study) without CHD at the baseline. Exclusion: cardiac rhythm disturbances (WPW syndrome, AF/flutter, wandering atrial pacemaker, SVT) | >10 years | Proportional hazard | Age, race, gender, education, hypertension status, systolic blood pressure, diabetes status, smoking, low-density lipoprotein cholesterol, high-density lipoprotein cholesterol, serum potassium and magnesium levels, cardiac rate, and medication use. | Risk for fatal CHD and any death were higher among those with PVCs compared with those without PVCs, regardless of the presence of CHD at baseline (No CHD group: RR 2.14, 95%CI 1.46-3.13, RR 1.48, CI 1.25-1.75, respectively; with CHD group: RR 2.12, CI 1.39-3.22, 1.74, CI 1.28-2.36, respectively) |
| Meine  2006 ^46^ | Quantify the incidence of recovery PVCs and correlate this finding to mortality and presence of ischemia in patients referred for exercise stress testing. | Cardiac catheterization within 180 days of a treadmill stress test. Exclusion: valvular or congenital heart disease or HF symptoms and currently not taking antiarrhythmic medications, and uninterpretable ECG due to a paced rhythm at rest | Median 4.6 years (max 10 years) | Logistic regression; Kaplan-Meier; cox proportional hazard | History of hypertension, Charlson co-morbidity Index, carotid bruits, cerebrovascular disease, peripheral vascular disease, diabetes, race, gender, third heart sound, age, sums stress (difference and rest scores), previous coronary bypass, and 3-vesel CAD | PVCs during recovery following stress test significantly predicted myocardial ischemia (OR 1.27, 95% CI 1.04-1.56) |
| Sajadieh 2006 ^47^ | Evaluate the prognostic significance of VPCs in an apparently healthy population. | Apparently healthy middle-aged and elderly population. Exclusion: 1) Manifest IHD (history of AMI, coronary revascularization, or angina pectoris; (2) manifest other cardiac diseases (CHF, valvular heart disease, congenital heart disease, atrial fibrillation or flutter, or medical treatment for any heart disease; (3) history of stroke; (4) left BBB, significant Q waves, and ST depression 1.0 mm; and (5) other significant or life-threatening diseases (cancer, acquired immune deficiency syndrome, cirrhosis hepatis, renal insufficiency requiring dialysis, or chronic lung disease requiring home oxygen therapy) | 53 months (51 - 55 interquartile) | Kaplan-Meier; cox proportional hazard | Age, gender, BP, smoking, diabetes mellitus, total cholesterol, body mass index, physical activity level, and silent ischemia | - Frequent PVC (≥30/h) was a significant predictor of combined events (HR 2.46, 95% CI 1.29 - 4.68) and CV events (HR 2.85, 95% CI 1.16 - 7.0). - Men with frequent PVCs of ≥30/hr (HR 3.15; 95% CI 1.57 - 6.30), and individuals with Framingham risk score >average with frequent PVCs of ≥30/hr (HR 2.53; 95% CI 1.16 - 5.54), had higher risk for the combined events   *NOTE:* Combined events = all-cause mortality or first AMI; Cardiovascular events = CV death or AMI |
| Engel  2007 ^102^ | Confirm the significance of PVCs and analyze the interaction between heart rate and PVCs. | Consecutive patients who had an ECG for any reason at the facility during study period. Exclusion: atrial fibrillation and paced rhythms | 5.5 years | Kaplan-Meier; cox proportional hazard | Age, gender, abnormal/normal ECG classification, and in- or outpatient status. | Presence of any resting PVCs was an independent predictor of mortality (HR 2.0; 95% CI 1.1–2.8) and CV mortality (RR 1.61, 95% CI 1.44–1.80) |
| Topaloglu 2007 ^48^ | Examine the effects of repetitive monomorphic PVCs on LV diastolic function. | Younger than 50 years of age who had symptoms of palpitations, dyspnea, or fatigue lasting for more than 1 year, in whom RVOT-PVCs (≥1000 ectopic beats/day) but with a normal LV systolic function. Exclusion: Significant obstructive CAD, presence of VT or atrial tachyarrhythmias, or history of thyroid disease, diabetes mellitus, and hypertension | NR | Multiple logistic regression | Age, total PVC count, LVEF, LVEDd, and LVESd. | Each 1% increment in the rate of daily PVCs was found to be associated with a 1.185 times increased risk of impaired LV relaxation (p=0.023) |
| Kanei  2008 ^103^ | Describe the prevalence and predictors of LV dysfunction in patients with frequent RVOT PVCs. | Frequent (≥10 PVCs per hour) RVOT PVCs. Exclusion: sustained supraventricular tachycardia, atrial fibrillation, or a pacemaker; also with history of IHD, structural heart disease, LV dysfunction with segmental wall motion abnormality on echocardiography, or with other apparent cause of LV dysfunction (i.e., alcohol, HIV) | NR | Logistic regression | Not provided | There was a higher prevalence of LV dysfunction among patients with RVOT PVCs >10,000/24 hr compared to those with <1,000/24hr and those with 1,000-10,000/24h (p=0.02). |
| Niwano 2009 ^49^ | Clarify the prognostic significance of frequent PVCs in asymptomatic or less symptomatic patients with normal LV function. | Frequent PVCs (>1000 beats/day) originating from RVOT or LVOT without any detectable heart disease | 5.6 ± 1.7 years | One-way ANOVA | Age, initial LVEF, PVCs/day, and PVC grade | - The PVC prevalence was negatively correlated ∆LVEF (p<0.001) and positively correlated with ∆LVDd (p<0.001) - PVC prevalence was an independent predictor of decrease LVEF by >6% (OR 85.4, p<0.01) - PVC prevalence cutoff point of 31,268 beats/day was significantly predicted LVEF reduction (AUC 0.724, sen 0.692, spec 0.929, p<0.0001). |
| Agarwal 2010 ^50^ | Examine the association between PVCs and incident stroke in a middle-aged population. | Participants from the general population (ARIC Study). Exclusion: cardiac rhythm disturbances (WPW syndrome, AF/flutter, wandering atrial pacemaker, SVT, not sinus rhythm), and missing information on prevalent history of stroke | 15 years | Cox proportional hazard | Age, gender, race, smoking status, heart rate, serum magnesium, serum potassium, prevalent CHD, diabetes, hypertension, LDL lipids, and HDL lipids | - Among individuals with no major risk factors for stroke, presence of PVCs (vs without PVCs) was associated with incident stroke (HR 2.09, CI 1.21-3.58) - Any PVCs (vs no PVC) among non-hypertensive was associated with embolic stroke of non-carotid origin (HR 3.48; CI 1.74-6.95) - Frequency of ≥4 PVCs/min compared to no PVCs was associated with incident stroke (HR 2.06, CI 1.24-3.42) - Risks for incident afib among those with any PVCs was higher than that in those with no PVCs (HR 1.56, 95% CI: 1.30, 1.87) |
| Baman 2010 ^51^ | Identify the critical PVC burden associated with cardiomyopathy. | Patients without CAD but with frequent PVCs referred for catheter ablation | 31 ± 20 months post ablation | ROC curve; proportional hazard | Not provided | - High PVC burden was independently associated with PVC-induced CMP (HR 1.12, CI 1.08-1.16) - A PVC burden of >24% best separated patients with impaired as compared with preserved LV function (sensitivity 79%, specificity 78%, AUC 0.89) |
| Hirose 2010 ^52^ | Investigate the association of PVCs and cardiac deaths in subjects without cardiovascular disease. | Apparently healthy individuals who had a baseline 12-lead ECG, without history of MI or stroke (Jichi Medical School (JMS) cohort study) | 11.9 years (SD not provided) | Cox proportional hazard | Age, BMI, systolic BP, total cholesterol level, HDL-cholesterol, and blood glucose | - In CV mortality, age-adjusted hazard ratios (HRs) with PVC were 3.73 (1.16-12.00) for males - HRs adjusted for multiple variables: 3.98 (1.21-13.00) for males |
| Le 2010 ^53^ | Study the prognostic value of PVCs at rest in patients with clinical HF referred for exercise treadmill testing. | Referred for exercise testing following a clinical presentation of HF | Median 6.2 years (range 5.5-6.7 years) | Kaplan-Meier; cox proportional hazard | Age, beta-blocker use, rest ECG findings, resting and peak HR, rest EF, maximal systolic BP, and exercise capacity | The presence of PVCs at rest was associated with a 5.48-fold (HR 5.48, p=0.004) increased risk of CV mortality |
| Cheriyath 2011 ^54^ | Prospective investigate to examine the relationionship between baseline ectopy and clinical cardiac events in a population-based sample without any history of cardiac disease or stroke. | General populations (ARIC Study) without CHD or stroke | Median 14 years | Kaplan-Meier; cox proportional hazard | Age, race, gender, education, smoking, BMI, LDL/HDL ratio, DM, hypertension, serum potassium, magnesium, HR, and use of heart rhythm medications | - Fully adjusted model showed that the presence of PVCs was significantly correlated with incident CHD (HR 1.24, 95%CI 1.02-1.50) - Fully adjusted model showed that presence of VPCs was significantly correlated with SCD and fatal CHD (HR [95%CI] 2.09 [1.22-3.56] and 2.18 [1.53-3.12], respectively) |
| Munoz 2011 ^55^ | Evaluate whether PVC characteristics are associated with LV systolic dysfunction. | Had radiofrequency catheter ablation for frequent and symptomatic PVCs, and had no other identified cause for cardiomyopathy | NR | Multivariate linear regression | History of palpitations, history of dizziness, PVC burden, nonsustained VT, PVC duration of ≥ 140 msec, fascicular PVCs, and multiform PVCs | - PVCs originating from the RV were associated with reduced LVEF at a PVC burden ≥10% - PVCs originating from the LV were associated with reduced LVEF at a PVC burden ≥20% - Independent predictors of a reduced LVEF: history of palpitations, presence of NSVT, PVC duration ≥140 msec, and nonfascicular PVCs |
| Agarwal 2012 ^56^ | Examine whether the presence of PVCs on a 2-minute ECG recording is associated with incident HF in individuals without prevalent HF. | General populations (ARIC Study) without prevalentHF. Exclusion: prevalent HF, missing information about baseline CHD and incident HF, and cardiac rhythm disturbances (WPW syndrome, AF/flutter, wandering atrial pacemaker, SVT, not sinus rhythm) | 15.6 ± 3.8 years | Cox proportional hazard | Age, gender, race, study center, education level, diabetes, systolic BP, hypertension medications, LDL and HDL cholesterol, BMI, current smoking, former smoking, pack-years of smoking, amount of ethanol use, heart rate, serum K+, serum Mg++ | - Presence of PVCs was associated with the incident HF (HR 1.84 (CI 1.41-2.41) - After multivariate adjustment, the HR of HF among those with any VPCs was 1.63 (1.36-1.96); remained significant after additional adjustment for LV mass (HR 1.59; 1.32-1.92) or medications (beta-blocker, anti-arrhythmic drugs, and calcium channel blockers); and after adjustment for incident CHD as a time-varying covariate (HR 1.71; 1.42-2.08) |
| Ofoma 2012 ^57^ | Evaluate the longitudinal relationship between premature cardiac contractions and incident ischemic stroke in a general population sample. | General population (ARIC Study). Exclusion: baseline history of stroke or CHD and individuals who developed subarachnoid or intracerebral hemorrhage during follow-up | 13 years | Cox proportional hazard | Age, race, gender, BMI, total cholesterol, diabetes, hypertension, smoking, and PAC/PVC predictor | Among normotensives, multivariable adjusted risk analysis showed that having PVCs was associated with increased risk of incident ischemic stroke (HR 1.69, 95%CI 1.02-2.78) |
| Yokokawa 2012a ^58^ | Correlate symptoms and symptom duration in the presence of CMP in patients with frequent PVCs. | Symptomatic and asymptomatic patients with frequent PVCs referred for catheter ablation but without structural heart disease | 95 ± 73 months post ablation | Logistic regression | Gender, PVC burden, duration of symptoms, and presence/absence of symptoms | PVC burden in asymptomatic patients was an independent predictor of impaired LV function (OR 2.1, 95% CI 1.2-3.6) |
| Yokokawa 2012b ^59^ | Determine the impact of the PVC QRS duration on reversible PVC-induced cardiomyopathy (rPVC-CMP). | Patients with frequent idiopathic PVCs referred for PVC ablation. Exclusion: CMP preceding frequent PVCs, presence of delayed enhancement in cardiac MRI, structural heart disease, and patients with CAD, valvular heart disease, or hypertensive heart disease | 46 ± 33 months | Logistic regression; ROC curve | Male sex, PVC burden, QRS width, epicardial origin of PVC, and symptom duration | - PVC-QRS width was predictive of rPVC-CMP (OR 1.03 for every 1-msec increase in QRS duration), independent of the PVC burden, symptom duration, sex, and PVC origin - PVC-QRS cutoff width of ≥150 msec best separated patients with and without rPVC-CMP (AUC 0.66; sensitivity 0.80; specificity 0.52) - Epicardial PVC site of origin was independently associated with rPVC-CMP (OR 2.9; P=.04) - PVC burden was predictive of rPVC-CMP (OR 1.04, p=.02). The PVC burden for developing rPVC-CMP is significantly lower in patients with a PVC-QRS width of ≥150 msec than in patients with a narrower PVC-QRS complex |
| Ban 2013 ^60^ | Determine the ECG and electrophysiological characteristics of frequent PVC-mediated LV dysfunction. | Frequent PVCs (burden >10%/day) with no significant structural heart disease. Exclusion: significant structural heart disease, including CAD, valvular heart disease, and congenital heart disease, and spontaneous or inducible sustained VT | 14 ± 14 months | Logistic regression; ROC curve | Gender, LVEF, LVEDd, LVESd, class I antiarrhythmics, amiodarone, PVC burden, non-sustained VT, and retrograde P wave | - The mean PVC burden (31 ± 11 vs. 22 ± 10%, P<0.001) and the presence of a retrograde P-wave following a PVC (64.3 vs. 30.3%, P=0.001) were significantly greater in those with LV dysfunction than in those with normal LV function - The cut-off PVC burden related to LV dysfunction was 26%/day (sens 70%, spec 78%) - Presence of retrograde P-wave following a PVC (OR 2.79, 95% CI 1.08–7.19, P=0.034) was independently associated with PVC-mediated LV dysfunction |
| Ephrem 2013 ^61^ | Evaluate the prognostic significance of both PVC frequency and morphology revealed on continuous ambulatory ECG monitoring. | Symptomatic patients (palpitations, syncope, and lightheadedness/near-syncope) referred for outpatient 24-hour ambulatory ECG monitoring who had at least one PVC during 24-hours of monitoring. Exclusion: ventricular couplets, triplets, or non-sustained VT | Median 2.3 years (range 2.0-2.6) | Kaplan-Meier; cox proportional hazard | Age, gender, heart rate, history of CAD, HTN, DM, dyslipidemia, diastolic or systolic HF, MI, smoking, alcohol consumption, family history of CAD, and use of aspirin, clopidogrel, statin, beta-blocker, ACEI/ARB, or CCB | Multiform PVCs were found to be a significant predictor of clinically adverse outcome independently of other covariates (HR 3.18, 95%CI 1.46-6.96), and remained significant after adjusting for CAD and prior MI (HR 3.05, 95%CI 1.39-6.70)  *NOTE:* Clinically adverse outcome: major adverse CV event (ACS, stroke, or all-cause mortality), or CHF. |
| Barutcu 2014 ^62^ | Investigate the effects of PVCs on LA function in patients without structural heart disease. | Frequent PVCs (>30 beats/hour) and without SHD. Exclusion: LV hypertrophy, LVEF <55 % or right ventricular dysfunction, valvular heart disease (including mild mitral regurgitation), Grade 3–4 hypertension, uncontrolled hypertension and diabetes patients using insulin, history of AF, renal disease, COPD, malabsorptive disease, systemic diseases, liver disease, thyroid dysfunction, hyperprolactinemia, and Cushing’s disease, endocrinopathy, neoplastic or metabolic disease, and using corticosteroids | NR | Mann-Whitney U/t-test; chi-square/Fisher’s exact; Spearman correlation | Not provided | - In patients with PVCs, LA dysfunction parameters LAd, LAVI and LAa) and LVED and LVSD diameters were found to be significantly higher, but LVEF was lower than in patients without PVCs - Global peak atrial longitudinal strain (PALS) and peak atrial contraction strain (PACS) were significantly lower; and time to peak longitudinal strain (TPLS) was significantly longer in the PVCs group. - Number of PVCs was positively correlated with TPLS, but negatively correlated with PALS and PACS |
| Cozma 2014 ^63^ | Assess LA remodeling in patients with frequent PVCs and explore the relationship between the PVC burden and LA shape and size. | Consecutive patients referred to clinic for persistent symptomatic PVCs in excess of 1,000/24-hr Holter. Exclusion: evidence of sSHD; hypertension for ≥5 years, persistent AF, sick sinus syndrome, CAD, significant comorbidities, and unexplained syncope | NR | Logistic regression | Age, sex, mean BP, LVEF, and LV end-diastolic diameter | Each 10% increase in PVCs was significantly associated with trapezoidal LA shape (OR 1.32, 95%CI 1.17-1.48) |
| Lee 2014 ^64^ | Investigate the relation of PVC burden, origin, QRS duration, and coupling interval with LV function and long-term survival in a large patient cohort with presence or absence of heart disease. | Diagnosed with PVCs | 3.4 years (SD 2.5) | Kaplan-Meier; logistic regression | Age, gender, presence of heart disease, dilated cardiomyopathy, symptoms of dizziness or palpitations, LV function, NSVT, heart rate, PVC origin, PVC coupling interval, PVC duration, antiarrhythmics, flecainide, propafenone, and ACEI/ARB | Greater coupling interval (OR 1.03, 95% CI 1.01-1.05) and PVC QRS duration (OR 1.02 [1.01-1.03]) were predictive for LVEF <50% |
| Park 2014 ^65^ | Investigate the impact of a high burden PVCs on early pathologic changes of cardiac performance in subjects with frequent PVCs and normal LVEF. | Frequent PVCs (>10/h) without SHD. Exclusion: congenital heart disease, LVEF of <55%, more than mild degree of valvular dysfunction, pericardial disease, IHD, cardiomyopathies, uncontrolled thyroid dysfunction, permanent pacemaker, or atrial fibrillation, or prescribed an antiarrhythmic drug, including β-blocker, within 6 months before enrollment | NR | Multiple linear and logistic regressions | LA volume index, LVEF, and systolic mitral annulus velocity | - There was independent association between frequent PVC and LAVI (OR 1.06, 95% CI 1.03–1.09) and LVEF (OR 0.92, 95% CI 0.89–0.96) - PVC burden was found to be an independent determinant of LAVI |
| Pol 2014 ^66^ | Determine whether PVC QRS duration measured before the onset of CMP would be a useful predictor of the subsequent development of CMP. | Underwent PVC ablation, had ≥10% PVC on preprocedural 24-hr Holter, and had PVC on ECG at least 6 month before ablation. Exclusion: structural or genetic heart disease, and history of sustained VT or sudden cardiac death | Median 14 mo (IQR 8-32 mo) | Multivariate logistic regression | Longer PVC QRS duration, longer conducted QRS duration, LV site of origin, and non-outflow tract site of origin | - Patients who developed PVC-induced CMP had significantly longer PVC QRS duration and sinus QRS duration - Longer PVC QRS duration (OR 2.94 [for each 10-msec increase in the baseline PVC QRS], 95%CI 1.36-6.55) and a non–outflow tract site of PVC origin (OR 14; 95% CI 1.55-126.84) were independent risk factors for LV dysfunction after multivariate analysis - PVC QRS duration of ≥153 msec best predicted development of PVC-CMP (sens 82%, spec 75%) |
| Qureshi 2014 ^67^ | Examine the prognostic significance premature contractions detected by a single 12-lead ECG in subjects without known cardiovascular disease. | Enrolled in the National Health and Nutrition Examination Survey (NHANES) III, > 20 years with good quality ECGs. Exclusion: known CVD, ECG evidence of MI, paced rhythms, or atrial fibrillation | 13 ± 4 years | Kaplan-Meier; cox proportional hazard | Age, sex and race/ethnicity, smoking status, systolic BP, BMI, BP medications, total cholesterol, diabetes mellitus, cancer and pulmonary disease (bronchial asthma and chronic obstructive pulmonary disease), ECG left ventricular hypertrophy, and corrected QT interval | In individuals > 65 years, presence of PVCs was associated with all-cause mortality (HR 1.36, 95%CI 1.04-1.76) |
| Yang 2014 ^68^ | Determine predictors of high-burden PVCs and, among high-burden PVC patients, predictors of HF. | At least a 20% PVCs burden | NR | Multivariable logistic regression | Age, sex, hypertension, CAD, creatinine, previous MI, CABG, CHF, and first-degree family history of sudden death | - High-burden PVC patients had a three-fold greater odds of HF (OR 3.15, 95%CI 1.28-6.50) - High-burden PVC patients had almost 10-fold greater odds of having a first-degree family history of sudden death (OR 9.97, 95%CI 1.78-60.8) |
| Agarwal 2015 ^69^ | Examine whether PVCs detected by the more conventional short-term routine ECG are associated with increased risk of ischemic stroke. | REGARDS study community-based participants without incident hemorrhagic stroke, prevalent stroke or transient ischemic attack. | 6 ± 2 years | Cox proportional hazard | Age, sex, race, geographic region, education level, prior heart disease, systolic BP, use of antihypertensives, left ventricular hypertrophy by ECG, atrial fibrillation, diabetes, current smoking, and use of warfarin and aspirin | PVCs were associated with a 38% increased risk of ischemic stroke (HR 1.38, CI 1.05–1.81) |
| Dukes 2015 ^70^ | Determine whether PVC frequency ascertained using a 24-h Holter is a predictor of a decrease in the LVEF, incident CHF, and death. | Enrolled in CHS study randomly assigned to 24-h ambulatory ECG (Holter) monitoring during initial assessment and who were part of the initial recruitment cohort. Exclusion: normal LVEF, as determined by the baseline echocardiogram, or with prevalent CHF | Median 13.7 years (range 8.0 - 18.2) for incident CHF; median f/u 15.2 years (range 9.6 - 18.4) for mortality | Cox proportional hazard | Age, sex, race, BMI, and history of hypertension, diabetes, CAD, beta-blocker use, atrial fibrillation, and number of VT episodes during Holter | - Patients in the upper quartile of PVC frequency (vs lowest quartile) had 3-fold greater odds of a 5-year decrease in LVEF (OR 3.10; CI, 1.42 to 6.77), and a 48% increased risk of incident CHF (HR: 1.48; CI: 1.08 to 2.04) during a median follow-up of 13 years - Patients in the upper quartile of PVC frequency (vs lowest quartile) had a 31% increased risk of death (HR: 1.31; CI 1.06 to 1.63) during a median follow-up of 13 years |
| Lin 2015 ^71^ | Evaluate the prognostic significance of PVC polymorphism in patients with apparently normal hearts. | Had 24-hour ECG monitoring due to palpitations, syncope, suspected arrhythmia, and clinical follow-up as clinically indicated, but otherwise with apparently normal hearts. Exclusion: prevalent sustained or non-sustained ventricular tachycardia (defined as reported history of tachycardia on baseline 12-lead ECG or on baseline Holter monitoring), permanent pacemaker (confirmed as a reported history of permanent pacemaker at their first study encounter on baseline 12-lead ECG or on baseline Holter monitoring), HF (confirmed by physician report, discharge summary, and echocardiography report), previous MI (confirmed by physician report or review of medical chart), history of ablation, valvular heart disease (confirmed by physician report or review of medical chart) and frequent PVC (>720/per day | 10 ± 1 years | Kaplan-Meier; cox proportional hazard | Age, sex, hypertension, DM, CKD, and use of hypertensive medication | - Patients with multiform PVC had an increased incidence of mortality (HR 1.642, 95% CI 1.327–2.031), hospitalization (HR: 1.196, 95% CI: 1.059–1.350), cardiovascular hospitalization (HR: 1.289, 95% CI: 1.030–1.613), new-onset HF (HF; HR: 1.456, 95% CI: 1.062–1.997), transient ischemic accident (HR: 1.411, 95% CI 1.063–1.873), and new-onset atrial fibrillation (AF; HR: 1.546, 95% CI: 1.058–2.258) - Patients with multiform PVC had a higher rate of mortality (HR: 1.231, 95% CI: 1.033–1.468) and all cause-hospitalization (HR: 1.147, 95% CI: 1.025–1.283) |
| Bas 2016 ^72^ | Assess the impact of circadian PVC variability on PVC-induced CMP. | Frequent PVCs referred for catheter ablation, with and without CMP | 3-6 months | Logistic regression | Asymptomatic, PVC burden, QRS duration of PVCs, pleomorphic PVCs, male sex, interpolation, symptom duration, and interquartile coefficient of PVC variation | - Patients with CMP had a higher PVC burden, less variability in circadian PVC distribution (coefficient of variability, CoV), and more frequent interpolated PVCs - Interquartile CoV cutoff value of ≤31% best distinguished patients with and without CMP (AUC 0.74; sens 66%; spec 81%; P = .0001) - PVC QRS duration (OR 1.086, 95% CI 1.014 - 1.16) and interquartile CoV <31% (OR 16.32, 95% CI 1.715 - 155.3) are independent predictors of PVC-induced CMP |
| Agarwal 2017 ^74^ | Examine interactions that might prove clinically useful in risk-stratifying patients with PVCs (large state-database, Healthcare Cost and Utilization Project [HCUP]).  *NOTE: PVC diagnosis was based on ICD-9 code 427.69* | Patients from the Healthcare Cost and Utilization Project (HCUP) California database. Exclusion: prevalent systolic or diastolic HF at the baseline, arrhythmogenic RV dysplasia, paroxysmal VT, valvular heart disease, and those with HF diagnosis during the same visit as the first diagnosis as PVCs | 5 years | Kaplan-Meier; cox proportional hazard | Age, gender, race, annual household income, and presence of CAD, hypertension (HTN), DM and AF | - Patients with PVCs exhibited an 80% excess risk of incidence systole HF (HR 1.8, 95% CI 1.8 – 1.9) - In patients aged <65 years (without HTN, DM, CAD, or AF), the diagnosis of PVCs increased the risk of incident HF by >6-fold (HR 6.5, 95% CI 5.5 – 7.7) |
| Lin 2017 ^75^ | Determine the predictive values of PVC frequency on mortality, CV hospitalization, and HF during long-term clinical follow-up. | Indication for 24-hour Holter monitoring (palpitations, syncope, and clinical follow-up). Exclusion: sustained VT, permanent pacemaker, or history of catheter ablation | 10 years (SD 1) | ROC curve; Kaplan-Meier; cox proportional hazard | Age, sex, HTN, CAD, DM, previous MI, valvular heart disease, HF, and taking ACEI/ARB or diuretic | - Optimal cut-off for PVC/24hr for predicting all-cause mortality was 12 PVCs/day (sens 58.3%, spec 59.8%, AUC 59.6%) - A propensity score matched analysis found that PVC >12/day was associated with new-onset HF (crude HR 1.38, CI 1.10 – 1.74), and CV-cause hospitalization (crude HR 1.24, CI 1.06 – 1.45) - PVC >12/day was associated with increased risk for all-cause mortality (adjusted HR 1.49, 95%CI 1.28-1.59) |
| Nguyen 2017 ^73^ | Investigate whether atrial or ventricular ectopy on a standard 10-second 12-lead ECG is associated with an increased risk of incident AF, HF, and mortality. | Participants from the CHS and ARIC studies. Exclusion: insufficient data, artificial pacing, wandering atrial pacemaker, or missing ECG data, prevalent HF and without baseline echocardiography for "myopathy" analyses | 10 years (SD not provided) | Kaplan-Meier; cox proportional hazard | Age, sex, race, study site, hypertension, diabetes mellitus, MI, CAD, beta-blocker, atrial fibrillation, study site, and baseline LVEF | - In univariate analysis, the presence of a PVC was associated with an increased risk of AF in the CHS participants (HR, 1.3, 95%CI, 1.1-1.6, p=0.007); with similar finding in ARIC cohort (HR, 1.9, 95%CI, 1.2-3.0, p=0.005) - After multivariable adjustment, there was 30% increased risk of incident HF in CHS participant (HR, 1.3; 95% CI 1-1.6), and 2-fold increased risk in ARIC study (HR 2.0, 95% CI 1.4 – 2.8) - There was an association between presence of a PVC with incident HF with systolic dysfunction (HR 1.8, 95% CI 1.2 – 2.5) - Over 5 years, a PVC on the baseline ECG was associated with approx. 3-fold greater odds of a reduction in LVEF (OR 2.8, 95% CI, 1.3-6.1) |
| Sheldon 2017 ^76^ | Compare clinical characteristics and outcomes of patients with pleomorphic vs monomorphic idiopathic PVCs. | Referred for ablation with a frequent idiopathic PVCs. Exclusion: SHD, no ambulatory ECG data, myocarditis, infiltrative heart disease, prior MI, or definitive late gadolinium enhancement (LGE) on cardiac MRI | 5.6 months (median f/u for ambulatory ECGs) | Multivariate logistic regression | Not provided | A cutoff of ≥156 non-predominant PVCs over 24hr best differentiated successful from unsuccessful ablation procedure (AUC 0.64, sens 56%, spec 74%) |
| Aviles-Rosales 2018 ^77^ | Find associations between exercise-induced PVCs, the occurrence of life-threatening ventricular arrhythmias and all-cause mortality in patients with cardiovascular disease. | Recognized CAD or idiopathic CMP (ICM), who initially performed stress testing as a part of their routine CV evaluation, and did not have VT | 14 years | Kaplan-Meier; cox proportional hazard | Diagnosis of idiopathic CMP or CAD, LVEF, digoxin, diuretic-espironolactone, antiarrhythmic, exercise tolerance, and life-threatening arrhythmia combined outcome | Patients with EiPVC showed an increased long-term risk of mortality (RR 2.1; 1.2-3.4) and life-threatening arrhythmia combined outcome (LACO) (RR 2.81; 1.9-4.3) |
| Bière 2018 ^78^ | Determine whether infrequent stress-induced right BBB morphology ventricular ectopy (SI-RBVE) provide prognostic information on CV outcomes. | Patients referred for SPECT | 4.5 ± 1.3 years | Cox proportional hazard | Age, history of HF, peripheral arterial disease, scar, LVEF <50%, LVEF <50% + stress-induced right BBB VE, and LVEF ≥50% + stress-induced right BBB VE | - There was a higher proportion of all-cause mortality in patients with SI-RBVE (23.4% vs 14.0%, p=0.021) - There was an interaction between SI-RBVE and LVEF. In patients with LVEF >50%, SI-RBVE was an incremental risk factor for mortality (OR 2.17, 95% CI 1.09-4.34, p=0.028) |
| Im 2018 ^99^ | Evaluate the association of frequent PVCs >10% and stroke-like symptoms without a prior diagnosis of stroke or TIA. | With or without documented PVCs, *and without* any history of CMP or valvular or congenital or IHD, hepatic or renal disease, acute cerebrovascular or cardiovascular events, major trauma or surgery, hyperthyroidism, uncontrolled hypertension, malignancy, connective tissue disease, acute/chronic inflammatory disease | 3.5 years | Kaplan-Meier; multivariate logistic regression | Age, atrial fibrillation, hypertension, LVH, E/E' (peak mitral flow velocity of the early rapid filling wave/early diastolic mitral annulus velocity), mitral regurgitation grade, and NT-proBNP | PVC >10% was found to be an independent risk factors for stroke-like symptoms without a prior diagnosis of stroke or TIA (OR 3.421, CI 1.092-10.726) |
| Li 2018 ^79^ | Evaluate the prognostic impact of baseline long-term continuous frequent PVC (CfPVC) burden among patients with an ICD. | Dual-chamber ICD capable of hourly PVC counting and home monitoring transmission, with daily average ventricular pacing percentage of <20%. Exclusion: single chamber ICD device, incapable of processing home monitoring, history of channelopathy and other inherited arrhythmias, and experienced outcomes measured during data collection period | Median 43 months (34-53 months) | ROC curve; Kaplan-Meier; cox proportional hazard | Age, gender, BMI, indication for ICD implantation, ICM, Non-ICM, stroke, valvular disease, DM, paroxysmal atrial fibrillation, hypertension, syncope, NYHA, LVEF, LVEDD, CfPVC percentage, and the use of amiodarone, β-blockers, ACEIs/ARBs and spironolactone | - CfPVC percentage ≥40% was an independent predictor of cardiac death (HR = 3.288; 95%CI = 1.720–6.283; p<0.001). - The best cut-off point of CfPVC percentage against ventricular arrhythmic events was 40% (AUC 0.703, p<0.001). This cut-off point means that the hourly mean PVC≥10 beats was accounted for 40% (24 days) of the 60-day monitor duration. |
| Ruwald 2018 ^80^ | Evaluate the association between 12-month ventricular ectopic burden and the risk of HF/death and malignant ventricular tachyarrhythmias in patients with cardiac resynchronization therapy with defibrillator (CRT-D) (from the Multicenter Automatic Defribrillator Implantation Trial with Cardiac Resynchronization Therapy (MADIT-CRT) | CRT-D from the MADIT-CRT study who had undergone both pre-implant and 12-month 24-hour Holter. MADIT-CRT criteria: depressed LV function (LVEF < 30%), prolonged QRS duration (>130 ms), ischemic (NYHA class I/II), or non-ischemic (NYHA class I) CMP and sinus rhythm at enrollment. Exclusion: within 3 months prior to enrollment, had undergone CABG, PCI, or had experienced an MI, or within 1 month prior to enrollment, had experienced atrial fibrillation | 2.23 ± 0.98 years | Kaplan-Meier; cox proportional hazard | Diabetes, LBBB QRS morphology type PVCs, male gender, hospitalizations 1 year prior to enrollment, glomerular filtration rate<60 ml/min/m2, and baseline LV end systolic volume index | - High ectopic burden (>10 PVCs/hr) was associated with a 2.8 fold increased risk of HF/death (HR 2.76, p<0.001) and VT/VF (HR 2.79, p<0.001) compared to patients with low ectopic burden - Patients with an unchanged high ectopic burden, and patients who experienced an increase in ectopic burden had a higher risk of HF/death and VT/VF, with 3-year cumulative incidences of 24%–25% (p<0.001, Kaplan-Meier curve) - Unchanged high PVC burden was associated with higher risk of HF/death (HR 2.9; CI 1.51 – 5.56) and VT/VF (HR 2.99; CI 1.66 – 5.37) - Increased PVC burden was associated with higher risk of HF/death (HR 2.97; CI 1.35 – 6.51) and VT/VF (HR 2.32; CI 1.09 – 4.97). |
| Su 2018 ^81^ | Explore the predictors for development of VT among patients with frequent PVCs. | Frequent PVCs (>30 beats/hour; and PVCs ranged 746 - 47083/24 h). Exclusion: Pulmonary heart disease, severe dysfunction of kidney and liver, rheumatic disease, tumor, and receiving treatment with digitalis, quinidine, or tricyclic antidepressant | NR | Multiple stepwise logistic regression | Potassium level, PVC burden, LVEF, couplets, and alcohol consumption | Extensive PVC burden (OR 1.07; 1.032-1.108) and PVC couplets (OR 33.984; 11.526-100.199) are associated with the occurrence of VT |
| Altıntaş 2019 ^82^ | Evaluate the relationship between PVC burden and LV ejection fraction (LVEF). | Adults with idiopathic PVCs >1,000 PVC/24h. Exclusion: less than 24 hr and/or inconclusive Holter recording, CAD, history of cardiac arrest, sustained VT, intracardiac defibrillator (ICD), second‐ or third‐degree AV block, sick sinus syndrome, permanent cardiac pacemaker, known or suspected etiology of cardiomyopathies including, ischemic, restrictive, hypertrophic, diabetic, arrhythmogenic, and non‐compaction before or on admission, genetic cardiac channelopathies, pericardial disease, myocarditis, all forms of atrial fibrillation, thyroid disorders, anemia, electrolyte disorders, chronic pulmonary disease, pulmonary hypertension, moderate‐to‐severe valvular heart disease, or pulmonary embolism | NR | Proportional odds logistic regression; generalized additive model | Age, sex, PVC burden, antiarrhythmic drug use, interpolated PVC, polymorphic PVCs, circadian variability, sinus QRS duration, PVC QRS duration, PVC coupling interval, LVOT, and RVOT site of origin | Increase in PVC burden (%), PVC QRS duration, and age are associated with decrease in LVEF. Decrease in LVEF become more prominent when PVC burden is >5%. |
| Yamada 2019 ^100^ | Validate the clinical significance of PVC burden on cardiac function, exercise capacity, and outcome in hospitalized patients with HF. | Hospitalized patients with decompensated HF, excluding patients with ACS and receiving hemodialysis | Median 2.3 years | ROC curve; Kaplan-Meier; cox proportional hazard | Age, NYHA class III/IV, LVEF, Holter‐based AF, diabetes, chronic kidney disease, anemia, receiving devise therapy, and the use of amiodarone. | - An increase of PVC burden (%/d) was associated with an increased risk of cardiac events (HR 1.036; CI 1.005‐1.068) - The optimal cutoff value of PVC burden (high/low) for predicting cardiac events at the 1‐year follow‐up was 0.145%/d (95% CI: 0.570‐0.714, p < .001, sens 64.2%, spec 59.0%, AUC 0.64)   *NOTE:* Cardiac events: ICD therapy, re-hospitalization due to worsening HF, or death. |

ACEI = angiotensin-converting-enzyme inhibitors; ACS = acute coronary syndrome; AF = atrial fibrillation; AMI = acute myocardial infarction; ANOVA = analysis of variance; ARB = Angiotensin II receptor blockers; ARIC = Atherosclerosis Risk in Communities; AUC = area under the curve; BHAT = Beta Blocker Heart Attack Trial; BMI = body mass index; CABG = coronary artery bypass graft; CAD = coronary artery disease; CCB = calcium channel blocker; CHD = coronary heart disease; CHF = congestive heart failure; CHS = Cardiovascular Health Study; CI = confidence interval; CKD = chronic kidney disease; CMP = cardiomyopathy; COPD = chronic obstructive pulmonary disease; CRF = chronic renal failure; CRT-D = cardiac resynchronization therapy with defibrillator; CVD = cardiovascular disease; DAVIT II = Danish Verapamil Infarction Trial II; DBP = diastolic blood pressure; DM = diabetes mellitus; ECG = electrocardiogram; EDVI = end-diastolic volume index; HCUP = Healthcare Cost and Utilization Project; HDL = high-density lipoprotein; HIV = human immunodeficiency virus; HR = hazard ratio; HR = heart rate; ICD = implantable cardioverter defibrillator; IHD = ischemic heart disease; LA = left atrium; LAa, LAd = left atrium area, diameter; LAD = left axis deviation; LAVI = left atrium volume index; LBBB = left bundle branch block; LDH = lactate dehydrogenase; LDL = low-density lipoprotein; LV = left ventricular; LVEDd = left ventricular end diastolic diameter; LVEF = left ventricular ejection fraction; LVESd = left ventricular end systolic diameter; LVOT = left ventricular outflow tract; MADIT-CRT = Multicenter Automatic Defibrillator Implantation Trial with Cardiac Resynchronization Therapy; MRFIT = Multiple Risk Factor Intervention Trial; MRI = magnetic resonance imaging; NHANES III = Third National Health and Nutrition Examination Survey; NR = not reported; NSVT = non-sustained ventricular tachycardia; NT-proBNP = N-terminal pro b-type natriuretic peptide; NYHA = New York Heart Association; OR = odds ratio; PAC = premature atrial contraction; PCI = percutaneous coronary intervention; PVC = premature ventricular contraction; RBBB = right bundle branch block; REGARDS = Reasons for Geographic and Racial Differences in Stroke; ROC = receiver operating characteristic; RR = risk ratio; RV = right ventricular; RVOT = right ventricular outflow tract; SBP = systolic blood pressure; SCD = sudden cardiac death; SD = standard deviation; SEM = standard error of the mean; SHD = structural heart disease; SPECT = single-photon emission computed tomography; TIA = transient ischemic attack; VF = ventricular fibrillation; VT = ventricular tachycardia.
